# Spillover and genome selection of the gut parasite *Nosema ceranae* between honey bee species

**DOI:** 10.3389/fcimb.2022.1026154

**Published:** 2022-10-11

**Authors:** Xiuxiu Wei, Jay D. Evans, Yanping Chen, Qiang Huang

**Affiliations:** ^1^ Honeybee Research Institute, Jiangxi Agricultural University, Nanchang, China; ^2^ USDA-ARS Bee Research Laboratory, BARC-East Building 306, Beltsville, MD, United States; ^3^ Department of Integrative Biology, The University of Texas at Austin, Austin, TX, United States

**Keywords:** *Nosema ceranae*, honey bee, multi-host parasite, selection, allele frequency

## Abstract

*Nosema ceranae* is a honey bee gut parasite that has recently spilled to another honey bee host through trading. The impact of infection on the native host is minor, which is substantial in the novel host. In this study, artificial inoculation simulated the parasite transmission from the native to the novel host. We found that the parasite initiated proliferation earlier in the novel host than in the native host. Additionally, parasite gene expression was significantly higher when infecting the novel host compared with the native host, leading to a significantly higher number of spores. Allele frequencies were similar for spores of parasites infecting both native and novel hosts. This suggests that the high number of spores found in the novel host was not caused by a subset of more fit spores from native hosts. Native hosts also showed a higher number of up-regulated genes in response to infection when compared with novel hosts. Our data further showed that native hosts suppressed parasite gene expression and arguably sacrificed cells to limit the parasite. The results provide novel insights into host defenses and gene selection during a parasite spillover event.

## Introduction

Host-parasite evolution involves reciprocal genetic changes between the interacting species (Brockhurst et al., 2014). When the parasite infects a novel host, high virulence is usually observed. The parasite spillover has been substantially facilitated by international trading. The honey bee is the most important commercial pollinator. The bee wax and fruit trading have led to the global spread of the bee parasite, causing substantial damage to the apicultural industry (Idrissou et al., 2019; Liu et al., 2021). *Nosema ceranae* is an obligate intracellular parasite that infects the epithelial cells of honey bee midgut (Higes et al., 2007). *N. ceranae* is a native parasite of the Asian honey bee *Apis cerana* and has successfully established infection in the novel honey bee species, *Apis mellifera* (Fries et al., 1996; Higes et al., 2006). Using a set of genetic markers, a high level of genetic diversity was found in *N. ceranae* (Gómez-Moracho et al., 2014; Pelin et al., 2015). However, minor variance was found among various geographically distinct regions, suggesting human-mediated gene flow. The infection exhibits high levels of virulence in the novel honey bee host, leading to impaired flying ability, reduced life span, and suppressed immune responses (Antunez et al., 2009; Dussaubat et al., 2013; Goblirsch et al., 2013; Gage et al., 2018). However, the impact of infection was minor in the native host. As the two honey bee species share habitats in Asia and Australia, it provides an ideal opportunity to use *A. mellifera*, *A. cerana*, and *N. ceranae* as model organisms to study the parasite spillover.

*N. ceranae* was recently proposed within *Vairimorpha* (Tokarev et al., 2020). To be consistent with previous literatures, we kept *N. ceranae*. This study aims to reveal the mechanism under the reported high virulence after the parasite spillover to the novel host. Accordingly, we quantified host and parasite gene expression, covering the entire proliferation cycle. Additionally, we investigated whether a subset of parasite genotype was favored leading to the high virulence.

## Results

### The native host showed high mortality following infection

**Figure 1:**
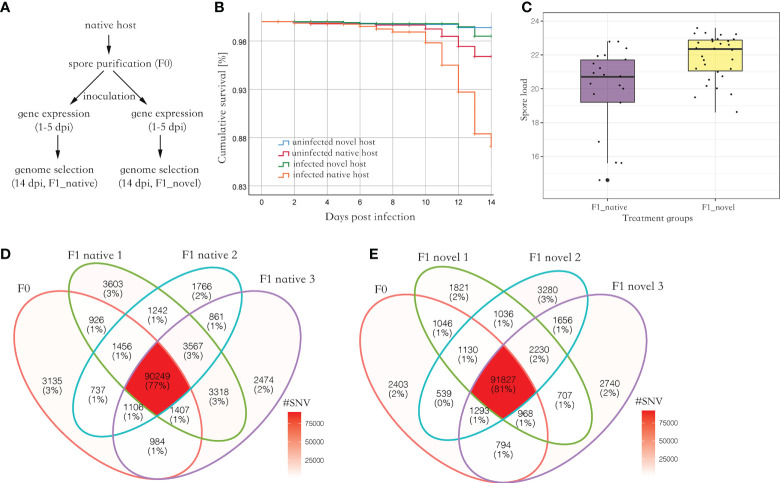
**(A)** The experimental design of this study. The spores were purified from the native host. Then the spores were inoculated to newly emerged honey bees from the native and novel host, respectively, to harvest produced spores. The gene expression (RNA-seq) of the infected bees was quantified from 1 to 5 dpi, covering a complete proliferation cycle of the parasite. Additionally, the SNVs along the parasite genome were quantified and compared between the parental and produced spores at 14 dpi. **(B)** The cumulative survival of honey bees. The native host infected by the parasite (F1_native) showed the highest mortality (Log Rank, adjusted *P* < 0.001). **(C)** The parasite spore load in the infected native and novel hosts. A significantly higher number of produced spores were found in the novel host compared with the native host (Kruskal Wallis test, *P* < 0.05). The y-axis represents the spore load which was log-transformed with base 2. **(D)** Venn diagram of SNVs identified in parental and produced spores in the native host. **(E)** Venn diagram of SNVs identified in the parental and produced spores in the novel host. Irrespective of the host species, at least 90% of parental SNVs were maintained in the produced spores. Additionally, over 50% of novel SNVs in produced spores were shared between the replicates in each host species, suggesting the mutation may not be random (Fisher’s exact test, *P* < 0.001).

Parasite spores were not found in the uninfected group. Among the experimental groups, uninfected novel host showed the highest survival (> 98%), which was not significantly different from the uninfected native host (Log Rank, adjusted *P* > 0.05). Comparatively, the infected native host showed the lowest survival (87%), which was significantly lower than the other three groups (Log Rank, adjusted *P* < 0.001) (**Figure 1B**). At 14 days post-infection (dpi), 5.1 million spores were found in the novel host, which was significantly higher than those found in the native host (Kruskal Wallis test, *P* < 0.05) (**Figure 1C**).

### Parasites initiated proliferation earlier in the novel than the native host

**Figure 2:**
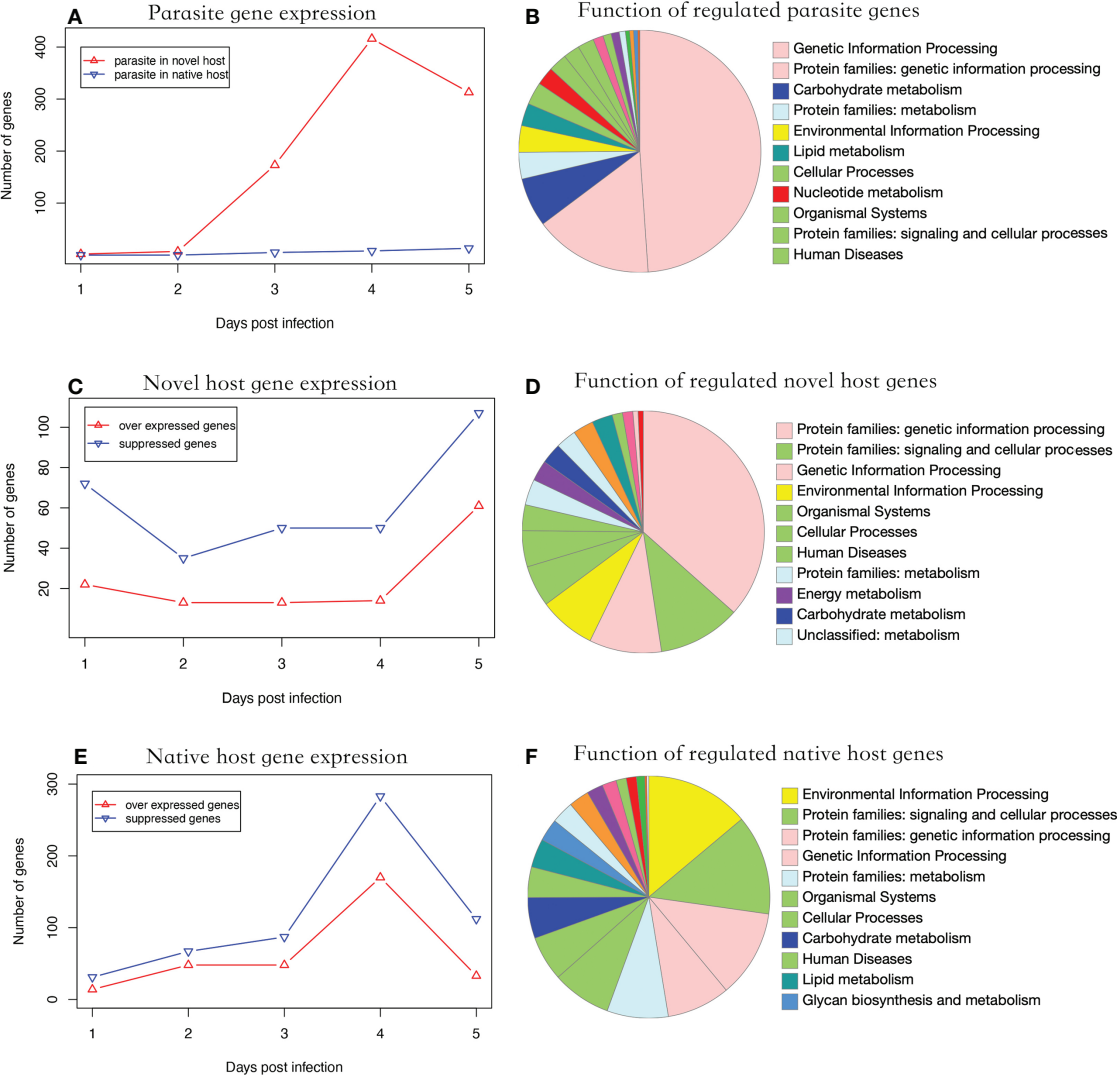
Functional analysis of regulated genes. **(A)** parasite gene expression profile in two hosts. The parasite showed higher gene expression levels in the novel than the native host (Fisher’s Exact Test, *P* < 0.001). **(B)** Putative function of significantly regulated parasite genes. **(C)** Number of significantly regulated genes of the novel host. The number of suppressed genes in the novel host was significantly higher than in over-expressed ones (Paired t-test, *P* < 0.01). **(D)** Putative function of the significantly regulated novel host genes. **(E)** Number of significantly regulated genes of the native host. The genes showed a suppressed expression pattern. **(F)** Putative function of the significantly regulated native host genes. In **(B, D, E)** the color of the functional category was used in global pathway maps and genome maps of KEGG. The functional category on the right was indicated in the pie chart in clockwise order.

During parasite proliferation, 762 genes were significantly over-expressed in parasites infecting the novel host, which is substantially higher than the 26 genes over-expressed in the native host (Fisher’s Exact test, *P* < 0.001) (**Figure 2A**). Significantly regulated genes were enriched in genetic information processing (**Figure 2B**). Parasite gene expression was detected as early as one dpi in the novel host. Comparatively, the infection was not detected until three dpi in the native host (**Figure 2A**). Additionally, 11 genes were over expressed at three time points when the parasite proliferated in the novel host, including a polar-tube protein (G9O61_00g021150). Using the genome as background, genes related to nuclear chromatin were significantly enriched in the parasite infecting the novel host (GO:0000790, adjusted *P* < 0.01).

### The native host showed more robust responses to parasite infection than the novel host

In the novel host, the number of suppressed genes was two-fold higher than over-expressed genes from one to five dpi (**Figure 2C**, Paired t-test, *P* < 0.01), which were enriched in genetic information processing (**Figure 2D**). In the native host, the number of suppressed genes was slightly higher than over-expressed ones but not statistically significant during the entire experimental period (**Figure 2E**, Paired t-test, *P* = 0.057). The significantly regulated genes were enriched in environmental information processing (**Figure 2F**). In honey bees, the number of genes significantly regulated after infection was significantly higher in the native host (719 genes) than in the novel host (342 genes; Fisher’s exact test, *P* < 0.001). Using the genome as the background, genes related to the cell cycle (GO:0000082, adjusted *P* < 0.001) were enriched in the novel host and those related to the regulation of transcription (GO:0006357, adjusted *P* < 0.001) were enriched in the native host.

### Minor impact of host species on the parasite genome selection

On average, 74,002,415 reads (150bps paired reads, over 1,000 times genome coverage) were aligned to the *N. ceranae* genome in a library, which accounted for 97.4% of total sequenced reads. In F0 spores, 99,999 Single Nucleotide Variants (SNVs) were identified. In the F1_native group, 105,768 (F1_native_1), 100,983 (F1_native_2) and 103,965 (F1_native_3) SNVs were identified (**Figure 1D**). Comparatively, 100,765 (F1_novel_1), 101,506 (F1_novel_2) and 100,772 (F1_novel_3) SNVs were identified in the F1_novel group (**Figure 1E**). Out of 99,999 SNVs in F0 spores, 90,249 and 91,827 SNVs were found in all three replicates in both native and novel hosts. Over 90% of the parental SNVs were found in spores produced by the infection, irrespective of the host species. The numbers of maintained SNVs were independent of host species (Fisher’s exact test, *P* > 0.05). The genome differentiation index (*F_ST_*) within species was slightly smaller than between the host species (**Table 1**). Within the produced spores, the native host showed greater genome differentiation than the novel host when compared with the parental spores. The parental and produced spores showed similar genome diversity (*π*) and *Watterson’s θ*, irrespective of the host species (**Table 1**). *Tajima’s D* was positive in all isolates. The coefficient S was quantified for the maintained SNVs from F0 to F1 spores. Among the top 1% loci with the highest allele frequency variation, the loci were highly congruent across replicates and the host species, which was significantly higher than random (Fisher’s Exact test, *P* < 0.001). Among those loci, 50 genes were identified, which were significantly enriched in ATP binding (GO:0005524, adjusted *P* < 0.001) and cell proliferation (GO: 0010971, adjusted *P* < 0.01).

**Table 1:** Population-genetic statistics for the three parasite isolates (Mean ± SE).

Parasites	*π*	*Watterson’s θ*	*Tajima’s D*	F1_native (*F_ST_*)	F1_novel (*F_ST_*)
F0	0.00851 ± 0.00007	0.00817 ± 0.00008	0.67 ± 0.01	0.0029 ± 0.0024	0.0015 ± 0.0009
F1_native	0.00855 ± 0.00007	0.00813 ± 0.00007	0.55 ± 0.01		0.0018 ± 0.0010
F1_novel	0.00845 ± 0.00007	0.00799 ± 0.00008	0.66 ± 0.01		

*π* indicates the genome diversity. *Tajima’s D* was corrected for the pooled population. Pairwise fixation index *F_ST_* was used for pairwise comparisons.

## Discussion

Understanding the co-evolution between hosts and their parasites is particularly important in ecology and conservation biology (Gómez and Nichols, 2013; Paplauskas et al., 2021). Most empirical and theoretical studies of host-parasite interactions were conducted in a paired host and parasite (Rabajante et al., 2016; Best et al., 2017; Ebert and Fields, 2020). In natural conditions, the parasite may infect and spill among multiple hosts (Bailey et al., 2009). Consequently, parasite transmission and diversity depend on the relative degree of host abundance and susceptibility (Hardy and Cook, 2010; Neiman and Fields, 2016). *N. ceranae* is an intracellular gut parasite of honey bees transmitted through the fecal-oral route. Transmission heavily depends on direct (such as trophallaxis among colony members) and indirect (via commonly visited flowers) contact among honey bees (Graystock et al., 2015). As a part of its range, *N. ceranae* has spilled from Asian honey bees to European honey bees in instances where the shifting dynamic depends on the relative abundance of the host species’ numbers (Hovestadt et al., 2019). Previous studies found that the infection showed a high virulence in the novel host (Eiri et al., 2015; Gage et al., 2018; Paris et al., 2018). In this study, we found that the parasite *N. ceranae* produced a significantly higher number of spores in novel hosts compared with the native host. This suggests that novel hosts cannot suppress parasite proliferation at the individual honey bee level, as we previously found for the colony level (Wu et al., 2022). Additionally, we found that native hosts showed higher mortality. Thus, native hosts might limit parasite growth by sacrificing infected nest members, a trait observed in a honey bee strain selected for *N. ceranae* tolerance (Huang et al., 2012). A similar phenomenon has also been observed in the native host towards the mite infestation, wherein infected larvae were sacrificed to limit mite reproduction (Page et al., 2016).

Using a set of markers, *N. ceranae* diversity has been reported to be higher within honey bee colonies than among neighboring colonies (Gómez-Moracho et al., 2014; Gómez-Moracho et al., 2015). Previously, we found that the genetic diversity of *N. ceranae* was substantially higher in habitats where the native and novel honey bee species co-exist (Ke et al., 2021). This study found minor disparities in genetic diversity variation across replicates. In our data, *π* was slightly higher than *Watterson’s θ*, leading to a small positive *Tajima’s D*, which suggests a small number of low-frequency alleles in all parasite isolates. The rare loci might have a strong tendency to be lost, and the genome was under balancing selection (Fu and Li, 1993; Stajich and Hahn, 2005). The fixation index *F_ST_* reflects the overall genome differentiation. In our data, the *F_ST_* was slightly higher within a species than between the two host species, which suggests host-specific adaptation shaped the genome evolution, even though the effect was not strong. In host-parasite studies, allele frequency fluctuation can provide insights into virulence and infection (Campbell et al., 2013; Papkou et al., 2019). In our study, the impact of host species on allele frequency variation was minor, which might reflect their close phylogenetic relationship.

The life cycle of *N. ceranae* is approximately four days (Higes et al., 2007; Gisder et al., 2011). In our data, the proliferation initiated earlier when infecting the novel host. As a result, more spores were found in the novel host. In the parasite genome, 2,280 genes were annotated. Out of those, 762 genes were significantly up-regulated when infecting the novel host, which was broadly involved in DNA/RNA replication. Notably, the polar tube protein was constantly up-regulated, which is essential to attach to the host cell membrane and is vital to initiate the infection (Han et al., 2017). When the expression level of the polar tube protein was suppressed, the number of produced spores was substantially reduced (Rodriguez-Garcia et al., 2018). After the inoculation, the native host strongly responded to the infection. It is known that *N. ceranae* suppresses apoptosis in infected honey bees and that bees with accelerated apoptosis are more tolerant of infection (Higes et al., 2013; Kurze et al., 2015). In our data, cell-cycle genes were suppressed in the novel host, suggesting that infected cells can’t initiate apoptosis to limit parasite proliferation. Thus, apoptosis may reflect an important strategy used by native hosts to limit *N. ceranae* infection.

## Materials and methods

### Host and parasite sources

The honey bee, *Apis cerana*, is the native host of the parasite *Nosema ceranae*, and the honey bee *Apis mellifera* is the novel host of the parasite *N. ceranae*. This study simulated parasite dispersal from native to novel host species by artificially inoculating the two honey bee species with *N. ceranae* spores. First, the parasite spores were purified from the native host, *A. cerana*. Then, the purified spores were used to inoculate the novel and native host separately to harvest produced spores by infection (**Figure 1A**). Overall, three sources of parasite spores were collected and analyzed, including parental spores isolated from native hosts (F0), spores produced by infection in the native hosts (F1_native), and spores produced in the novel hosts (F1_novel). Additionally, the parasite gene expression (RNA-seq) in the two hosts was quantified from one to five dpi, which covers a complete parasite proliferation cycle.

### Parasite isolation, inoculation, and sample collection

To harvest sufficient number of spores, 200 honey bee foragers were randomly collected from four *A. cerana* colonies that were heavily infected with *N. ceranae* in the experimental apiary. Midguts of the honey bees were dissected and homogenized to isolate *N. ceranae* spores using centrifugation (Fries et al., 2013). The spores were further purified using Percoll gradient centrifugation and the species status of *N. ceranae* was confirmed by species-specific primers using conventional PCR (Chen et al., 2013; Fries et al., 2013). The pooled spores represented local parasite diversity and served as a common source for inoculations.

Frames with emerging brood were removed from colonies of the native (*A. cerana*) and novel host (*A. mellifera*), which were kept in an incubator (34 ± 1°C, 60% relative humidity). For each honey bee species, 150 newly emerged honey bee workers were individually fed with 2 µl of sucrose solution containing 10^5^ *N. ceranae* spores isolated from the native host. An additional 150 newly emerged native and novel host honey bee workers were fed with 2 µl sucrose solution as an uninfected control. During the experiment, cohorts were divided into three cups containing 50 bees each and maintained on 50% sucrose solution *ad libitum* in the incubator. Dead bees were recorded and removed daily. Three bees were collected from one to five dpi from each cup at 24h intervals. The midgut was dissected for RNA extraction with TriZol and sequenced using Illumina Hiseq 2000. At 14 dpi, the remaining honey bees were harvested for spore counting. The spores of the same species were pooled for DNA extraction using CTAB (cetyl trimethylammonium bromide) (Chen et al., 2013), which were also sequenced using Illumina Hiseq 2000. For each treatment, three replicates were performed.

### Gene expression and *SNV* analysis

For genetic diversity analysis, a DNA library for F0 spores, three DNA libraries for F1_native spores (F1_native_1, F1_native_2 and F1_native_3) and three DNA libraries for F1_novel spores (F1_novel_1, F1_novel_2 and F1_novel_3) were sequenced. DNA sequencing reads were aligned to the *N. ceranae* genome (Ncer 3.0, GCA_004919615.1) using BWA with default parameters (Li and Durbin, 2009; Huang et al., 2021). The SNVs were identified and annotated using the Picard-GATK-SNPEFF pipeline (Van der Auwera et al., 2013). The SNVs found in F0 spores but not in F1 spores were defined as lost SNVs. The SNVs found in F1 spores but not F0 spores were defined as novel SNVs. The SNVs found in both F0 and F1 spores were defined as maintained SNVs. For the RNA-seq analysis, three RNA libraries for the uninfected native host, three RNA libraries for the uninfected novel host, three RNA libraries for the infected native host, and three RNA libraries for the infected novel host were sequenced per day from 1 to 5 dpi. The RNA reads were aligned to the parasite (*N. ceranae*, Ncer 3.0, GCA_004919615.1), the native host (honey bee *A. ceranae*, HAv3.1, GCA_003254395.2), and the novel host (honey bee *A. mellifera*, ApisCC1.0, GCA_002290385.1) with Hisat2 with default parameters (Kim et al., 2015; Diao et al., 2018; Wallberg et al., 2019). The variance of the three replicates was used to calculate the within-group variance to determine significantly regulated genes using EdgeR (Robinson et al., 2010). The protein sequences of significantly regulated genes were quired to NCBI non-redundant database and KEGG to infer putative biological function and pathways (Kanehisa and Goto, 2000). GO terms were retrieved using EggNOG-mapper, and enrichment analysis was performed using TopGO (Alexa and Rahnenfuhrer, 2021; Cantalapiedra et al., 2021). In total, 7 DNA and 60 RNA libraries were sequenced and analyzed.

### Population genetic analysis and statistics

*Watterson’s θ*, genome diversity *π*, and corrected *Tajima’s D* values were calculated using Popoolation (Kofler et al., 2011a). The fixation index *F_ST_* was calculated using Popoolation2 (Kofler et al., 2011b). Survival was analyzed with the Kaplan-Meier procedure using SPSS and adjusted for FDR using R (R Core Team, 2013). The spore load variance was analyzed using the Kruskal Wallis test using R (R Core Team, 2013). Paired t-tests were used to compare the number of up and downregulated genes with R (R Core Team, 2013). As the common parental spores were inoculated to harvest produced spores, one sample t-test was used to compare the number of SNVs in parental and produced spores using R (R Core Team, 2013).

## Data availability statement

The datasets presented in this study can be found in online repositories. The names of the repository/repositories and accession number(s) can be found below: https://www.ncbi.nlm.nih.gov/, PRJNA784016; https://www.ncbi.nlm.nih.gov/, PRJNA820988.

## Author contributions

XW conducted the experiment. QH designed the experiment. XW, JE, YC, and QH organized the manuscript. All authors contributed to the article and approved the submitted version.

## Funding

This research was funded by the initiation package of Jiangxi Agricultural University (050014/923230722) and the National Natural Science Foundation of China #32060778.

## Acknowledgments

We appreciate Dr. Lizhen Zhang for technical support and Prof. Nancy Moran for revising the manuscript.

## Conflict of interest

The authors declare that the research was conducted in the absence of any commercial or financial relationships that could be construed as a potential conflict of interest.

## Publisher’s note

All claims expressed in this article are solely those of the authors and do not necessarily represent those of their affiliated organizations, or those of the publisher, the editors and the reviewers. Any product that may be evaluated in this article, or claim that may be made by its manufacturer, is not guaranteed or endorsed by the publisher.
